# The role of acid-sensitive ion channels in panic disorder: a systematic review of animal studies and meta-analysis of human studies

**DOI:** 10.1038/s41398-018-0238-z

**Published:** 2018-09-07

**Authors:** Laiana A. Quagliato, Rafael C. Freire, Antonio E. Nardi

**Affiliations:** Laboratory of Panic and Respiration, Institute of Psychiatry, Federal University of Rio de Janeiro, Rua Ataulfo de Paiva 135s. 609, Rio de Janeiro, 22440-901 Brazil

## Abstract

Acid-sensitive ion channels, such as amiloride-sensitive cation channel (ACCN), transient receptor potential vanilloid-1 (TRPV1), and T-cell death-associated gene 8 (TDAG8) are highly related to the expression of fear and are expressed in several regions of the brain. These molecules can detect acidosis and maintain brain homeostasis. An important role of pH homeostasis has been suggested in the physiology of panic disorder (PD), with acidosis as an interoceptive trigger for panic attacks. To examine the effect of acid-sensitive channels on PD symptoms, we conducted a systematic review and meta-analysis of these chemosensors in rodents and humans. Following PRISMA guidelines, we systematically searched the Web of Science, Medline/Pubmed, Scopus, Science Direct, and SciELO databases. The review included original research in PD patients and animal models of PD that investigated acid-sensitive channels and PD symptoms. Studies without a control group, studies involving patients with a comorbid psychiatric diagnosis, and in vitro studies were excluded. Eleven articles met the inclusion criteria for the systematic review. The majority of the studies showed an association between panic symptoms and acid-sensitive channels. PD patients appear to display polymorphisms in the *ACCN* gene and elevated levels of TDAG8 mRNA. The results showed a decrease in panic-like symptoms after acid channel blockade in animal models. Despite the relatively limited data on this topic in the literature, our review identified evidence linking acid-sensitive channels to PD in humans and preclinical models. Future research should explore possible underlying mechanisms of this association, attempt to replicate the existing findings in larger populations, and develop new therapeutic strategies based on these biological features.

## Introduction

Acid-sensitive channels are highly related to the expression of fear. In mice, deleting or inhibiting acid-sensitive ion channels (ASICs), such as ASIC1a, transient receptor potential (TRP) vanilloid-1 (TRPV1), or proton-sensing G protein-coupled receptors, such as T-cell death-associated gene 8 (TDAG8) can render the animal less fearful to conditioned and/or unconditioned fear^1–4^.

Fear is produced by CO_2_ inhalation, which also generates autonomic and respiratory responses that can evoke panic attacks in individuals with panic disorder (PD)^5^. For this reason, CO_2_ is frequently used as a biological challenge and biomarker for PD^6^. In addition to CO_2_, other agents such as lactate can cause pH shifts and evoke panic attacks^7^. Furthermore, neuroimaging studies suggest the presence of dysregulated acid-base buffering^8^ and increased plasma and brain lactate responses to metabolic challenges in patients with PD^9^.

Brain extracellular pH is a fundamental signal for regulating homeostatic arousal, such as in behavior and breathing. In the intact brain in vivo, interstitial pH generally ranges from 7.1 to 7.25, but this balance can be endangered by numerous conditions, including metabolic acidosis, inflammation, and hypoventilation, in which pH can drop to 6.5^10^. Monitoring systems, such as molecular acid sensors, deal with these challenges by detecting harmful acidosis, and initiating appropriate emergency reactions, thereby limiting any resulting tissue damage^11^. Among the molecular acid sensors, ASICs, also known as amiloride-sensitive cation channel (ACCN), TRPV1, and TDAG8 are the most extensively studied^11^.

ASIC1a, which can be activated at pH 7.0^12^, is reportedly involved in the amygdala’s control of learning mechanisms^13^. This function is also related to the TRPV1 channel. TRPV1-expressing neurons are activated by threatening stimuli^1^, and this channel does not begin to open until pH reaches 6.4^14^. The pH range for TDAG8 stimulation and signaling is 5.2–5.7^15^, and its deficiency leads to attenuated CO_2_-evoked freezing in animal models^2,3,16^.

Taken together, these observations support the hypothesis of defective homeostatic acid-base regulatory systems in PD. We thus aimed to review the evidence that acid-sensitive channels are involved in PD pathophysiology and to identify gaps in the literature to inform future research.

Primary outcome:

- Verify the presence of an association between acid-sensitive channels and PD symptoms.

Secondary outcomes:

- Ascertain whether deletions or antagonisms of acid-sensitive channels decrease PD symptoms.
- Identify single nucleotide polymorphisms (SNPs) in acid-sensitive channel genes related to PD.
- Elucidate the molecular basis linking acid-sensitive channels and PD.

## Materials and methods

### Data sources

We searched Web of Science, Medline, Pubmed, Scopus, Science Direct, and SciELO up to 10 June 2018. The references cited in the systematically searched articles were checked manually. In an effort to avoid publication bias, the search also included non-English language studies and gray literature (for example, conference abstracts). The search used a broadly structured strategy based on the Problem, Intervention, Comparator, Outcome, Setting (PICOS) framework, where the problem was PD symptoms, the intervention/exposure was polymorphisms, antagonisms, and deletions of acid-sensitive channels, the comparison was absence of PD symptoms, the outcome was an improvement or worsening of PD symptoms, and any type of study design was allowed. Search terms included various combinations of terms for panic and acid-sensitive channels, such as “panic disorder” OR “panic attacks” AND ASIC, “acid-sensing ion channel”, ACCN, ACCN2, ACCN1, “amiloride-sensitive cation channel”, TDAG8, “GPR65 protein, human”, “GPCR25 protein, mouse”, “TDAG8 protein, rat”, “transient receptor potential vanilloid-1 ion channel”, TRPV1, “TRPV cation channels”, “two-pore domain K+”, K2P, “ionotropic purinoceptors”, and P2X. The full search strategy is available in Supplementary Material 1.

### Study selection

Studies were selected for data extraction and analysis based on the following inclusion criteria: (1) original research studies in humans and/or animals associating acid-sensitive channels with PD symptoms; (2) in human studies, subjects met PD criteria based on a conventional psychiatric classification system, while control subjects did not meet criteria for PD; (3) in animal studies, only studies with recognized preclinical models of PD^17^, such as elevated T-maze and/or escape behavior induced by electrical/chemical stimulation of the periaqueductal gray matter, and a control group were included. Escape behavior was characterized by jumps and crossings. The following exclusion criteria were used in the search: (1) participants had a comorbid or additional psychiatric diagnosis that would exclude or confound PD; (2) studies that lacked a baseline condition or control group; (3) human studies with prepubertal participants; and (4) in vitro studies. The same search criteria were used to identify non-human and human studies.

### Data extraction and quality score

The following variables were extracted from all the studies: authors, year of publication, subject characteristics in the affected and control groups, and characteristics of experiments involving acid-sensitive channels. The main outcome change in panic symptoms in humans and non-humans was the presence of a polymorphism, deletion, or antagonism in acid-sensitive channels. The current review was conducted according to Preferred Reporting Items for Systematic Reviews and Meta-Analyses (PRISMA) guidelines^18^ (Supplementary Material 2). Quality assessment used the OHAT Risk of Bias Tool (Supplementary Material 3).

### Statistical analyses

A meta-analysis was performed when there were at least three studies investigating antagonism of acid channels in a specific human polymorphism. Animal studies were not statistically analyzed since their outcomes presented high heterogeneity. The threshold of a minimum of three studies for the meta-analysis was selected so that there were at least two replication attempts of the original finding^19^. Statistical analyses of the extracted data were conducted using the Comprehensive Meta-Analysis Program, version 3.

Pooled odds ratios (ORs) with their 95% confidence intervals (95% CIs) were calculated to evaluate the strength of the association between the ACCN2 rs685012 polymorphism and PD symptoms based on the allele model (C vs. T). Statistical heterogeneity between eligible studies was evaluated by using the Cochran’s Q statistic and *I*^2^-test. A *p*-value above 0.1 or *I*^2^ below 50% indicated substantial homogeneity across studies^20^. Therefore, the fixed-effects model using the Mantel–Haenszel weighting method was selected^21^ to perform the meta-analysis. Otherwise, the DerSimonian and Laird random effects model was chosen^22^. The study intended to assess publication bias using funnel plot techniques, Begg’s rank test, and Egger’s regression test, as appropriate, given the known limitations of these methods^23^.

## Results

**Fig. 1:**
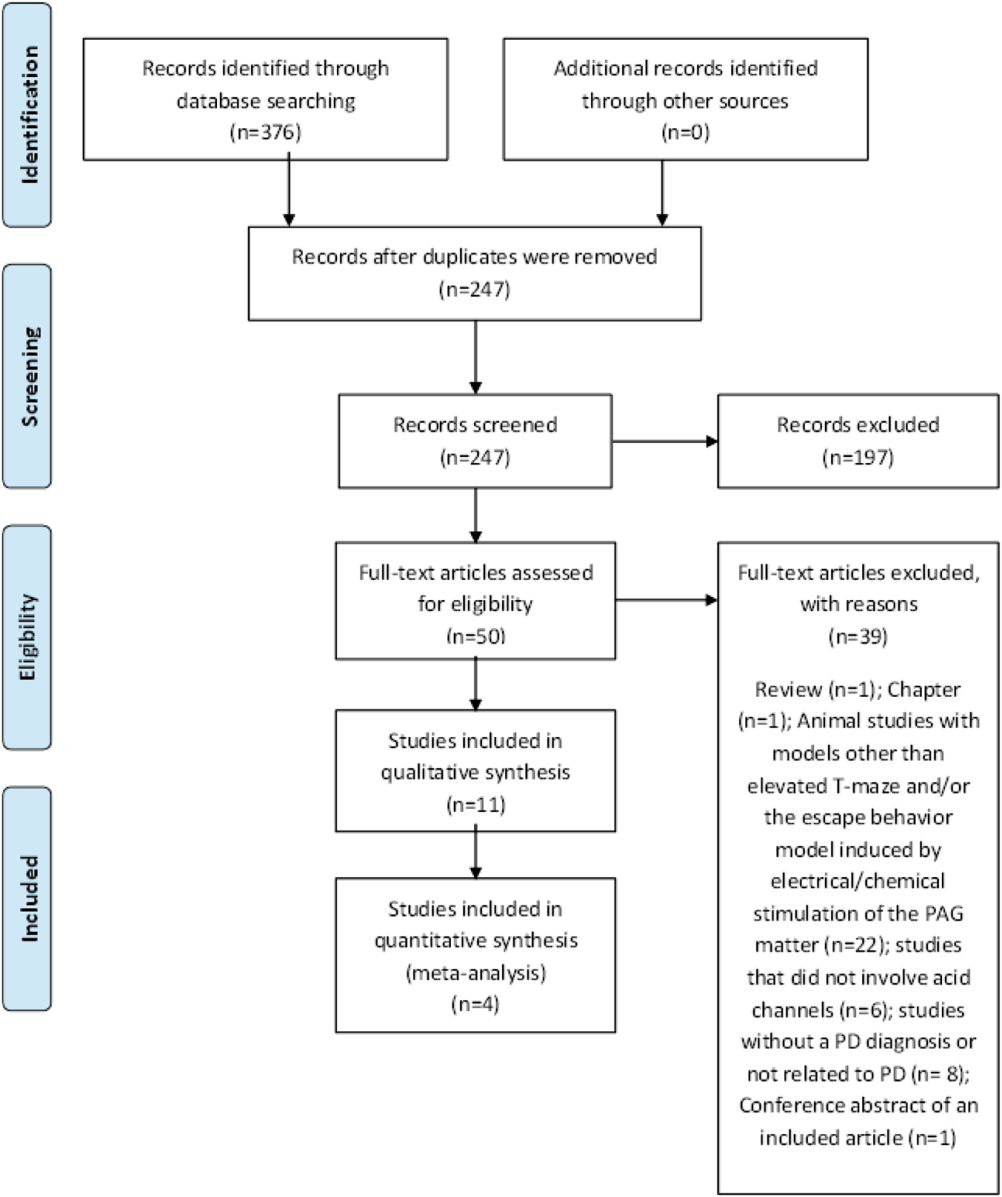
PRISMA Flow diagram. Flowchart of the systematic review and meta-analysis according to the PRISMA guidelines

The use of PRISMA guidelines and a systematic search of electronic databases yielded a total of 376 studies. No additional studies were identified through manual searching of references. After elimination of duplicates, 247 titles were reviewed, of which 197 were excluded. In all, 50 full texts were reviewed, of which 11 met the inclusion criteria for our systematic review (Fig. 1). Table 1 summarizes the studies associating acid-sensitive channels with panic-like symptoms in non-humans, and Table 2 summarizes the findings in humans.

**Table 1:** Non-human studies on acid-sensitive channels and panic-like symptoms

Study	Species	Strain	Sex	Weight	Acid channel	Control (*n*)	Panic (*n*)	Drugs	Area	*F*-value	*p*-value	Outcome
Almeida-Santos et al.^24^	Rats	Wistar	Male	220–240 g	TRPV1	9	7	Capsazepine	dlPAG	*F* (8,33) = 2.81	<0.05	Blockade of TRVP1 receptors in the dlPAG decreases escape responses in animals exposed to the ETM test
Batista et al.^25^	Rats	Wistar	Male	250–350 g	TRPV1	14	29	Arachidonoyl-serotonin	IV route	*F* (3,39) = 1.07	0.37	No effect of blockade on escape behavior
Casarotto et al.^26^	Rats	Wistar	Male	300–330 g	TRPV1	9	20	Capsazepine	dlPAG	*F* (3,25) = 16.58	<0.05	Capsazepine increases the threshold of electric current required to induce a panic-like response
dos Anjos et al.^27^	Rats	Wistar	Male	230–270 g	TRPV1	8	8	6-I-CPS	Ventromedial hypothalamus	NA	>0.05	Pretreatment with 6-I-CPS prevented escape behavior
Lisboa et al.^28^	Rats	Wistar	Male	230–270 g	TRPV1	35	22	Capsazepine	dlPAG	*F* (1,53) = 10.6	<0.005	Capsazepine reduced flight reactions

*TRPV1* transient receptor potential vanilloid receptor-1, *6-I-CPS* 6-iodonordihydrocapsaicin, *dlPAG* dorsolateral periaqueductal gray, *IV* intravenous, *ETM* elevated T-maze, *NA* not available

**Table 2:** Studies on acid-sensitive channels in panic disorder patients

Study	Design	Country	Patients	Controls	Dominant ethnicity	Diagnostic criteria	Experiment	Candidate genes /RNA	SNP	Genotypes	Patients (%)	Controls (%)	*p*-value	Outcome
Gugliandolo et al.^29^	Case control	Italy	71	100	Sicilian	SCID	TaqMan	ACCN2	rs685012	Allele C	64.7	47	0.030	PD was associated with the SNP rs685012
										Allele T	35.2	53		
Leibold et al.^30^	Case control	The Netherlands	183	107	Caucasian	MINI	TaqMan	ACCN2	rs10875995	Allele C	49.18	51.4	0.032	T allele of rs10875995 was associated with higher fear scores in PD
									rs685012	Allele T	50.81	48.59	0.061	
										Allele C	49.7	52.33		
										Allele T	88.5	47.66		
Smoller et al.^31^	Case control	US	414	846	Caucasian	SCID	MassArray system	ACCN2	rs685012	Allele C	61.83	53.9	0.011	PD associated was associated with the SNPs rs685012 and rs10875995
										Allele T	39.13	45.8		
									rs10875995	Allele C	58.68	51.88	0.46	
										Allele T	41.30	48.10		
Hettema et al.^32^	Case control	US	188	188	Caucasian	SCID	TaqMan	ACCN2	rs685012	Allele C	34.5	89.3	0.077	No significant associations were found
										Allele T	6.54	10.6		
Gregersen et al.^33^	Case control	Denmark	305	969	Caucasian	ICD-10	Sequenom platform	ACCN1	rs9915774	Allele A	12	17	0.006	A nominally significant allelic association was observed between PD and rs9915774
										Allele G	88	83		
Strawn et al.^34^	Case control	US	15	17	Caucasian	SCID	TaqMan	TDAG8 mRNA		NA	NA	NA	0.008	Higher expression of TDAG8 mRNA in PD

*SCID* structured clinical interview for *DSM*, *MINI* mini international neuropsychiatric interview, *ICD-10* international statistical classification of diseases and related health problems 10th revision, *SNP* single nucleotide polymorphism, *ACCN2* amiloride-sensitive cation channel 2, *ACCN1* amiloride-sensitive cation channel 1, *TDAG8* G protein-coupled receptor T-cell death-associated gene 8, *NA* not available

### TRPV channels

We found no human study on TRPV channels that met the search criteria. Five studies assessed how TRPV1 antagonism affected escape behavior in male rats^24–28^. The majority of studies reported that TRPV1 blockers decreased escape responses in animals^24,26–28^. Three out of five studies evaluated the effects of capsazepine, a TRPV1 receptor antagonist, in the dorsolateral periaqueductal gray (dlPAG). These studies were conducted in different animal models and demonstrated that blockade of TRPV1 receptors in the dlPAG decreased escape responses (*p* < 0.05)^24,26,28^. One study investigated the effects of the TRPV1 antagonist 6-iodonordihydrocapsaicin (6-I-CPS) in the ventromedial hypothalamus and confirmed that blocking this receptor decreased jumping and crossing responses^27^ (*p* > 0.05). Another study found no effect of TRPV1 blockade on escape behavior^25^.

### ASIC/ACCN channels

**Fig. 2:**
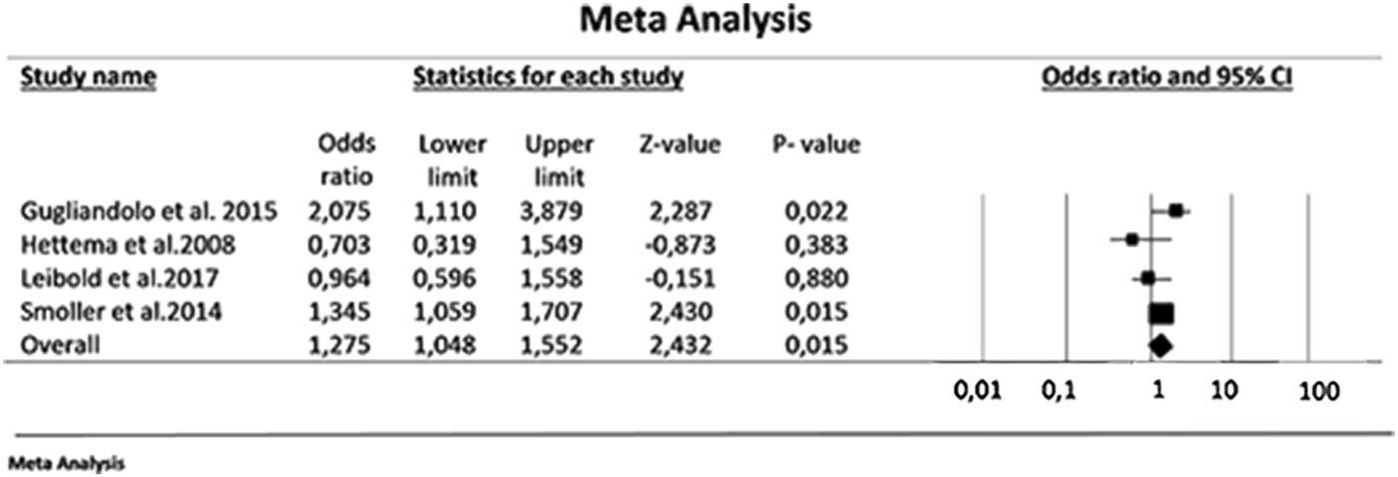
Meta-analysis of the strength of the association between the ACCN2 rs685012 polymorphism and PD symptoms. The C allele of the rs685012 polymorphism was associated with a significant increase in the risk of PD (summary effect size = 1.275; *p* = 0.015)

A meta-analysis of four studies involving 1981 participants (742 PD patients and 1239 controls) evaluated ACCN2, the human homologue to the rodent ASIC1a^29–32^. These studies assessed the C and T allele in the rs685012 SNP of the *ACCN2* gene and showed a significant increase in C allele in PD patients compared to its presence in controls (effect size: 1.275; 95% CI: 1.048–1.552; *p* = 0.015; Fig. 2). Although no significant heterogeneity was detected (*p* = 0.112), the inconsistency was moderate (*I*^2^ = 50.01%). Publication bias was not assessed as there were inadequate numbers of included studies to properly assess a funnel plot or to perform more advanced regression-based assessments. Interestingly, the C allele of SNP rs685012 in the *ACCN2* gene was related to early-onset PD and prominent respiratory symptoms in one study^31^.

In addition, we examined the association with ACCN2 variants using neuroimaging measures of amygdala structure and function. The PD risk allele C at rs10875995 was associated with increased amygdala volume as well as task-evoked amygdala reactivity to angry and fearful faces^31^. Furthermore, the ACCN2 rs10875995 T/T genotype was related to higher fear scores in PD patients^30^. Examination of SNPs within the *ACCN1* gene revealed a nominal association between this gene and PD^33^.

### TDAG8

A recent study evaluating blood samples of 15 individuals 15–44 years of age with a diagnosis of PD and 17 healthy controls showed significantly higher TDAG8 mRNA expression in mononuclear cells from PD patients than in those from controls (*p* = 0.008), as well as an association between TDAG8 mRNA expression and PD symptom severity (*p* < 0.001). There are concerns related to the generalizability of these findings, since this pilot study evaluated only a small sample of PD patients. Nevertheless, elevated TDAG8 mRNA expression was found even in young patients close to the onset of their illness, and a trend suggested a relationship between this receptor and treatment response in PD patients previously treated with antidepressants (*p* = 0.08)^34^.

## Discussion

Our review shows that acid-sensitive channels are related to PD and panic symptoms. Specifically, there was evidence of a reduction in panic-like symptoms after TRPV1 blockade in the dlPAG and hypothalamus in the majority of studies (4 out of 5) in rodents^24,26–28^. These studies were performed with injections directly targeting-specific brain regions, while the only study that failed to show any association between TRPV1 antagonism and panic symptoms used intravenous administration of arachidonoyl-serotonin^25^. Although systemic injection may be more appropriate for testing a potential therapeutic effect, the pharmacokinetic properties of the drug are important to consider. For example, arachidonoyl-serotonin is catalyzed by a cytochrome P450 enzyme that is widely expressed^35^, potentially limiting the availability of the drug in the tissue and contributing to the negative findings of the study.

A meta-analysis involving four studies identified the C allele of the rs685012 SNP in the *ACCN2* gene as a significant risk factor for PD symptoms^29–32^. Interestingly, the C allele of SNP rs685012 in the *ACCN2* gene was related to PD cases with prominent respiratory symptoms^31^. The C allele of rs10875995 in the *ACCN2* gene was associated with increased amygdala volume, as well as task-evoked amygdala reactivity to fearful and angry faces^31^, while the T/T genotype was associated with higher fear scores in PD patients^30^. This variation could potentially be attributed to differences in endophenotypes, since the C allele was shown to be associated with PD diagnosis and amygdala volume and function, i.e., largely anxiety-related endophenotypes, whereas the T allele was associated with experimentally provoked fear/panic sensations, i.e., specific fear-related endophenotypes^30^.

Another case control study investigated TDAG8 mRNA in PD and demonstrated higher mRNA expression in PD patients than in controls^34^. Taken together, these results suggest that acid-sensitive channels are related to PD symptoms. Nevertheless, further studies are needed to confirm these findings and determine the potential mechanisms associating acid-sensitive channels with PD.

### A hypothesis on the mechanism of action of acid-sensitive channels in the central nervous system (CNS) and its potential effect on PD symptoms

The underlying mechanism of the effect of acid-sensing ion channels on PD remains to be fully established. However, this mechanism could be elucidated by rodent models of panic-like behaviors. In rats, both electrical and chemical stimulation of the dlPAG induces fight and flight behaviors, along with cardiovascular changes^36^. Since these responses resemble those observed in humans with PD, stimulation of this region has been suggested to serve as an experimental model of panic attack^37^. Likewise, the elevated T-maze (ETM) has been proposed as an animal model to study panic-related behavior^17^. In this model, a rat must perform a one-way escape test in which the animal is positioned at the end of one of the open arms, and the escape latency is measured three times^38^. The latter is associated with the escape response and has been associated with PD^38^. In our review, studies evaluated the effects of a TRPV1 receptor antagonist in the dlPAG and demonstrated that TRPV1 receptor blockade in this region decreased escape responses. TRPV1 inhibition in the ventromedial hypothalamus (where electrical stimulation leads to tachycardia and panic in humans^39^) also decreased panic-like behavior in animals.

Findings in translational rodent models of panic could provide information on potential ion channels and receptors that may contribute to the pathophysiology of PD in humans. Clinical studies over the years have shown that an imbalance in acid-base homeostasis may exist in PD patients, thus pointing to the relevance of pH sensing as well as the underlying circuits that contribute to pathophysiological responses. As an interoceptive stimulus, CO_2_ inhalation can evoke panic attacks^5^. In humans, CO_2_-sensitivity lies on a continuum^40^, with PD subjects being highly sensitive to low CO_2_, while healthy volunteers only experience panic-like symptoms at higher concentrations^41,42^. A study in twins demonstrated high concordance for CO_2_ sensitivity^43^, suggesting a genetic etiology for this interoceptive stimulus. Our findings suggest that SNP in the *ACCN* gene is associated with PD symptoms, while the C allele of SNP rs685012 in the *ACCN2* gene was more prevalent in respiratory-subtype PD patients^31^. Nevertheless, PD does not develop in all individuals with CO_2_ hypersensitivity. Therefore, a combination of genetic and environmental factors may determine hypersensitivity to CO_2_ and PD symptoms^44,45^.

Environmental factors as well as various cognitive processing errors also likely play a part in the development of panic attacks^46^. Even if a threat or danger is perceived by a cognitive process, the pituitary gland produces adreno-corticotropic hormone, which in turn stimulates the adrenal cortex to produce the hormone cortisol^46^. Drug-naive PD patients show higher baseline cortisol levels than controls^46^. Cortisol may significantly increase the acid secretion capacity of H^+^-ATPase at the cellular level, which could contribute to systemic acidosis in PD patients^47^.

Systemic acidosis may also occur through a combination of CO_2_ with water, a reaction that may be responsible for the panicogenic effects of CO_2_^48^. Evidence indicates that ASIC1a channels located in the amygdala detect a reduced pH arising from increased CO_2_ or from direct injection of acid, initiating a fear response^2^. However, studies in patients with Urbach–Wiethe disease indicate that the amygdala is not required for the expression of panic and fear in response to CO_2_ inhalation^49^, suggesting that distinct chemosensors in other brain regions may be responsible for fear and panic responses in response to interoceptive stimuli.

**Fig. 3:**
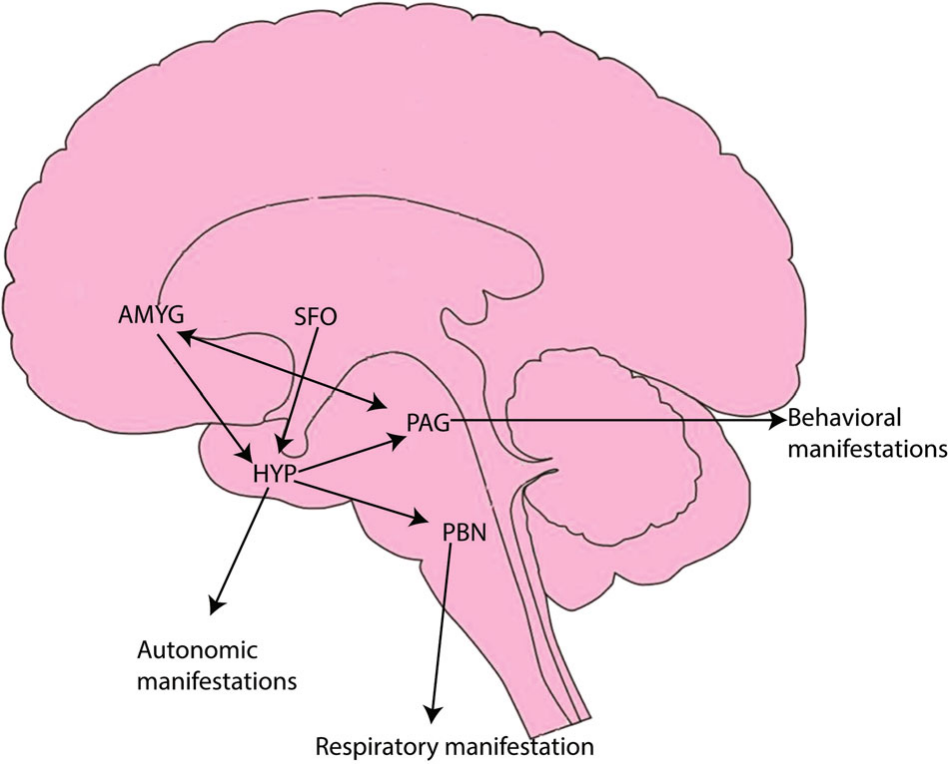
Acidosis sensed by acid channels may be translated as autonomic, respiratory, and behavioral symptoms of a panic attack. Respiratory manifestations could be controlled by the PBN via direct inputs from the hypothalamus and indirect inputs from the SFO. The hypothalamus may regulate autonomic expression through inputs from the amygdala and SFO. Additionally, the hypothalamus, amygdala, and PAG could be involved in behavioral symptoms. AMYGD amygdala, HYP hypothalamus, PAG periaqueductal gray, SFO subfornical organ, PBN parabrachial nucleus

In addition to the amygdala, acid-sensitive circuits are present in other brain regions potentially relevant to PD, including the bed nucleus of the stria terminalis, periaqueductal gray (PAG), hypothalamus, and circumventricular organs^9,50,51^. Acidosis sensed by acid channels may be translated to autonomic, respiratory, and behavioral symptoms of a panic attack^52^. Respiratory symptoms may be controlled by the parabrachial nucleus (PBN) via inputs from the hypothalamus^53^ and indirectly from the subfornical organ (SFO) ^54^, while the amygdala, PAG, and hypothalamus may regulate the autonomic and behavioral manifestations of panic^55^ (Fig. 3).

The SFO, which is a sensory circumventricular organ (CVO), has access to systemic and CNS compartments for the maintenance of homeostasis^56^. Recent studies associate the SFO with panic-like responses to intravenous lactate^57^ and CO_2_^3^. PD patients are also susceptible to the induction of panic attacks not only by CO_2_ but also by systemic administration of a variety of agents, such as lactate, cholecystokinin, and norepinephrine. Many of these agents do not cross the blood-brain barrier easily^57^. Therefore, regions lacking a blood-brain barrier can be exposed to these circulating substances and in turn stimulate other areas, such as the amygdala or the dorsomedial hypothalamus, thereby eliciting a panic response. Such a mechanism involving CVOs could also provide a single unifying explanation for the existence of multiple, apparently unrelated, agents that appear to induce panic attack in PD patients^57^.

The acid-sensitive channels TRPV1^58^, ASIC^59^, and TDAG8^3^ are expressed in microglial cells in several regions of the brain, including CVOs^8,51^. Microglia, innate immune cells of the CNS, are recruited in physiological responses to homeostatic fluctuations, transforming from a resting to a proinflammatory activated state^60,61^. Extracellular acidification induces rapid alteration in microglial morphology^62^, suggesting a potential role of microglia in the effects of acidotic stimuli. Furthermore, recent findings associated alterations in the immune system with PD^63^. Considering the close relationship between acid-sensitive channels and microglia, an active engagement of these cells in the detection of an acidotic pH threat is tempting to be considered as a potential mechanism in the genesis of panic attacks.

Additionally, acid-sensitive channels can be classified as proton channels and may contribute to the maintenance of the microglial membrane potential and phenotypes^64^. Panic attacks triggered by agents such as caffeine, cholecystokinin, and norepinephrine could be explained by modifications in microglial ion currents^64^, altering the membrane potential of the cells and potentially transforming microglia from a resting to an active state^64^. However, the relationship between PD symptoms and acid chemosensors in microglia has not been fully investigated but is an important future direction for understanding the contribution of microglia to PD pathophysiology.

### Translational applicability of acid-sensitive channel mechanisms: from an animal model to PD patients

While there is a clear need for more studies in primates addressing the issues discussed above, our findings that pharmacological blockade of acid channels in animal models of PD implies that these channels may contribute to PD treatment. PD has been treated primarily with drugs that have anxiolytic properties, including benzodiazepines and selective serotonin reuptake inhibitors^65^. Nevertheless, 20–40% of patients do not achieve full remission with the recommended medicine^65^. Thus, new therapeutic targets and drug development for PD patients should be further investigated.

In humans, dlPAG stimulation produces emotional and autonomic responses strikingly similar to those of panic attacks, whether spontaneous or provoked by intravenous infusions of lactate^66^. In addition, dlPAG-evoked panic-like behaviors are attenuated by clinically effective panicolytics given at doses and regimens similar to those given for panic therapy^67^. One animal model of PD is the escape behavior induced by electrical/chemical stimulation of dorsal portions of the PAG matter. After stimulation of the dlPAG, a vigorous reaction is observed, with piloerection, miosis, vertical jumps, and strong flight reactions represented by an increase in locomotion and average speed^17^. Another preclinical model for PD is the ETM animal test. The ETM test assumes that a panic attack is a reaction to a proximal threat when no threat is present. Therefore, this test summons neural mechanisms that underpin proximal defense, which can be investigated by experimentally analyzing the escape task in the ETM^17^.

Findings in translational rodent models of panic could provide information that may contribute to our understanding of the pathophysiology of this disorder in humans. Our review shows that pharmacological blockade of TRPV1 channels decreases panic symptoms in animal models with injections directly targeting-specific brain regions. In humans, antagonists of TRPV1 channels are being studied in clinical trials on osteoarthritis and atopic dermatosis, conditions where there is a pH imbalance^68,69^. In none of these trials does the drug need to enter the brain. Thus, future studies are needed to investigate drugs that potentially block TRPV1 channels and cross the blood-brain barrier.

Blockade of amiloride ion channels may also be a promising therapeutic target for PD, since a SNP in the *ACCN* gene is related to PD symptoms in humans. The C allele of SNP rs685012 in the *ACCN2* gene is related to PD cases with prominent respiratory symptoms^31^. In addition, PD patients with the respiratory subtype of the disorder are more sensitive to CO_2_ challenge^70^. Mirroring human studies of PD, animal studies have shown that inhalation of CO_2_ evokes fear behavior in mice, an effect that is reduced by deletion or blockade of ASIC1a, which is homologous to the ACCN human gene, within the amygdala. Overexpression of ASIC1a in the amygdala is sufficient to trigger CO_2_-induced fear behavior^2^. The ACCN2 allele associated with both PD and amygdala volume is associated with increased amygdala reactivity to emotional faces, a phenotype linked to PD^31^. The observed association between allelic variants and amygdala reactivity has been suggested to potentially reflect enhanced sensitivity to reduced pH secondary to neuronal activity that mediates the processing of emotional stimuli^31^. Therefore, ASIC antagonists such as amiloride are being studied and have shown promise for acidosis-associated conditions, such as stroke, migraine, pain, spinal cord injury, and multiple sclerosis^71^. Recent studies have shown neuroprotective effects of amiloride and corroborated the efficacy of this agent for alleviating pH-associated pathophysiology^71^.

A role for pH and chemosensory mechanisms in panic physiology is increasingly appreciated on the basis of the recent data from animal studies. However, the degree to which these systems may be targeted by psychopharmacologic interventions in PD is still unexplored. Thus, the lack of studies related to pH-modulating therapies is of public health significance, given that the therapeutic options for PD are limited.

### Limitations and strengths

There are a number of limitations to this systematic review and meta-analysis. Importantly, as part of our search criteria in preclinical data, we only included studies associating acid-sensitive channels with ETM and/or escape behavior tests. Although no animal model to date has perfectly mimicked panic symptoms, the relationship between these models and PD is recognized in the literature^17^. Other preclinical models such as elevated plus maze and predator encounter-based models could also evaluate some panic symptoms such as freezing or fear conditioning^17^. However, we chose not to expand our search criteria, since these models are also related to other anxiety disorders such as generalized anxiety disorder and post traumatic stress disorder. Although these disorders are commonly comorbid with PD, we opted not to include preclinical models evaluating exteroceptive threat response systems since our main goal in this review was to evaluate internal triggers and interoceptive chemosensory pathways of particular relevance to PD. In addition, preclinical models investigating nociception and long-term potentiation were also excluded. Supplementary Material 4 lists the full-text articles that were excluded and the reasons for their exclusion.

Regarding human studies, although PD is highly comorbid with other conditions, especially depressive disorder, we excluded articles involving participants with a comorbid or additional psychiatric diagnosis and/or those that were prepubertal. Nevertheless, some comorbidities may have been unintentionally included in our review, since even the best-designed studies may not properly assess all comorbidities. Although this exclusion may have limited the number of studies included in the review, this exclusion was justified since the variety of acid channels investigated can be expressed by microglia, the innate immune cells of the CNS. Evidence has shown that the majority of mental health disorders can be related to neuroinflammation^72^. Inflammation per se may contribute to an acidic environment. Based on changes in the microglial microenvironment, the investigated acid channels can display a variety of complex spatiotemporal patterns^64^. The same is true for age-related alterations in the microglial microenvironment^64^. We thus excluded studies with participants that had a comorbid psychiatric diagnosis or were prepubertal, since they might present a different pattern of acid-sensitive channels in the microglia.

Finally, one general limitation to the included studies was that only a few patients were evaluated for polymorphisms in genes for acid-sensitive channels, which greatly limits the statistical power to detect actual SNP associations with phenotype.

In addition, the preclinical studies only included male rats. Research has shown higher numbers of acid-sensing ion channels in female mice than in males^73^. There are also differences in acid-sensitive channels in the brains of mice when compared to those in the brains of rats^74^. Thus, the extrapolation of findings on acid-sensitive channels in animal models of panic-like symptoms should be viewed with caution, and studies in female animals and non-rat species are needed.

Although pH imbalance is commonly accepted to be linked to PD and acid-sensitive channels are thought to play a role in PD pathophysiology, no authors to date have systematically summarized the literature to determine whether this relationship is backed by the totality of evidence. The current article reports the results of a systematic review examining acid-sensitive channels in human and animal studies. To our knowledge, this is the first systematic review and meta-analysis that has evaluated acid channels and PD based on PRISMA guidelines and after evaluating the quality of the included articles. One strength of the review is that by including multiple databases and following PRISMA guidelines, relevant studies were unlikely missed. The findings indicate that TRPV1, TDAG8, and ACCN channels are related to PD pathophysiology. However, more studies are needed to corroborate these results, which could represent an important step towards elucidating PD pathophysiology and contributing to new therapeutic options for the disorder.

### Future directions

A number of steps are necessary to fully understand the associations between acid-sensing ion channels and the neurobiology of PD. Antagonists of acid-sensitive channels in humans are a promising therapeutic target for PD. To date, there have been no clinical studies in PD patients on interventions targeting control of pH imbalance or acid-sensitive ion channel blockers. Nevertheless, ASIC antagonists such as amiloride have shown promise for other acidosis-associated conditions, such as pain and multiple sclerosis^71^. Therefore, clinical trials evaluating PD symptoms and acid channel blockade are needed, since acid channels could represent new therapeutics options for this disorder. Moreover, future genetic studies of SNPs in genes for acid-sensitive channels should consider replicating the initially reported findings through well-powered studies.

Much of the research to date has focused on the dysregulation of central fear circuitry, including the limbic network, which involves connections between the amygdala, anterior cingulate cortex, and PAG, during panic symptoms^67^. The potential role of areas devoid of a blood-brain barrier in PD is important to investigate, especially given their connectivity to downstream sites responsible for the expression of behavioral and physiological responses.

Preclinical animal models are also needed to evaluate pH chemosensory interoceptive stimulus processing. These replicas could elucidate the interaction between different pH chemosensory molecules in the brain. A variety of sensory mechanisms in distinct areas can provide a highly sensitive pH detection system, which can be relevant to PD. Such animal models are also important for testing new drugs. Additionally, studies are needed on the effect of gender and species.

While there is significant evidence for the role of pH homeostasis and impaired acid-base buffering in patients with PD^8^, not all data support the link between acidosis and panic attacks, since not all panicogens cause acidosis, and hyperventilation (which produces alkalosis) can also produce panic attacks. Future studies are required to further clarify these inconsistencies.

More fundamental mechanistic research is essential if we are to truly understand the role that acid plays in the context of PD. Acid chemosensors on microglia could provide further insights into the integration of interoceptive pH fluctuations leading to behavioral and respiratory arousal. Future studies focusing on the crosstalk among acid chemosensors and inflammatory and neuromodulatory functions and the relationship among them will provide further insight into the pathophysiological mechanisms of PD.

## Conclusion

According to this systematic literature review, acid-sensitive channel antagonists decreased escape behavior in preclincal animal models of PD. In humans, an acid-sensitive channel SNP was shown to be associated with PD, PD symptoms, and a subtype of PD patients. Acid-sensitive channels may play an important role in the pathophysiological mechanisms of PD and provide a promising therapeutic target for the disorder. Future research should explore possible mechanisms underlying this association, attempt to replicate existing findings in larger populations, and aim to develop new therapeutic strategies based on these biological features.

## Electronic supplementary material

Supplementary Material 1

Supplementary Material 2

Supplementary Material 3

Supplementary Material 4
