# Supplementary material for: The role of acid-sensitive ion channels in panic disorder: a systematic review of animal studies and meta-analysis of human studies
Source: Transl Psychiatry. 2018 Sep 7;8:185. doi: 10.1038/s41398-018-0238-z (PMC6128878; doi:10.1038/s41398-018-0238-z)
Supplement: Supplementary file 1 — Supplementary Material 1 [file 41398_2018_238_MOESM1_ESM.doc]

Search Strategy

| **Search** | **Query** |
| --- | --- |
| 1 | “panic disorder” |
| 2 | “panic attacks” |
| 3 | ASIC or “acid sensing ion channel” or ACCN or ACCN2 or ACCN1 or “amiloride-sensitive cation channel” |
| 4 | TDAG8 or “GPR65 protein, human” or "GPCR25 protein, mouse" or "TDAG8 protein, rat" |
| 5 | TRPV1 or “transient receptor potential vanilloid-1 ion channel” or "TRPV Cation Channels" |
| 6 | “Two-pore domain K+” or K2P |
| 7 | “ionotropic purinoceptors” or P2X |
| 8 | 1 OR 2 |
| 9 | 3 OR 4 OR 5 OR 6 OR 7 |
| 10 | 8 AND 9 |
