# Supplementary material for: The role of acid-sensitive ion channels in panic disorder: a systematic review of animal studies and meta-analysis of human studies
Source: Transl Psychiatry. 2018 Sep 7;8:185. doi: 10.1038/s41398-018-0238-z (PMC6128878; doi:10.1038/s41398-018-0238-z)
Supplement: Supplementary file 3 — Supplementary Material 3 [file 41398_2018_238_MOESM3_ESM.docx]

| **Study** | **comparison group** | **confounding** | **missing outcome data** | **exposure characterization** | **outcome assessment** | **outcome reporting** |
| --- | --- | --- | --- | --- | --- | --- |
| Gugliandolo et al. 2015 | ++ | ++ | ++ | ++ | NR | ++ |
| Hettema et al. 2008 | + | ++ | ++ | ++ | NR | ++ |
| Leibold et al.2017 | ++ | ++ | ++ | ++ | ++ | ++ |
| Smoller et al.2014 | ++ | ++ | ++ | ++ | NR | ++ |
| Gregersen et al. 2012 | ++ | + | ++ | ++ | NR | ++ |
| Strawn et al.2017 | ++ | ++ | ++ | ++ | NR | ++ |

Table 1- Quality Assessment case-control studies

Table 2- Quality Assessment experimental animal studies

| **study** | **randomization** | **allocation concealment** | **identical experimental conditions** | **performance bias** | **bliding** | **missing outcome data** | **exposure characterization, confidence** | **outcome assessment** | **outcome reporting** |
| --- | --- | --- | --- | --- | --- | --- | --- | --- | --- |
|  |  |  |  |  |  |  |  |  |  |
| Almeida-Santos et al.2013 | + | ++ | ++ | ++ | ++ | ++ | ++ | ++ | ++ |
| Batista et al.2017 | NR | ++ | ++ | ++ | ++ | ++ | ++ | ++ | ++ |
| Casarotto et al. 2011 | + | ++ | ++ | ++ | ++ | ++ | ++ | ++ | ++ |
| Lisboa et al.2012 | + | ++ | ++ | ++ | NR | ++ | ++ | + | ++ |
| dos Anjos-Garcia et al.2016 | + | ++ | ++ | ++ | NR | ++ | ++ | + | ++ |
